# Unilateral Nevoid Telangiectasia After Pregnancy

**DOI:** 10.5826/dpc.1104a119

**Published:** 2021-10-01

**Authors:** Astrid Herzum, Claudia Micalizzi, Aurora Parodi

**Affiliations:** 1 Department of Dermatology, Di.S.Sal., University of Genoa, San Martino Polyclinic Hospital IRCCS, Genoa, Italy

## Case Presentation

**Figure 1:**
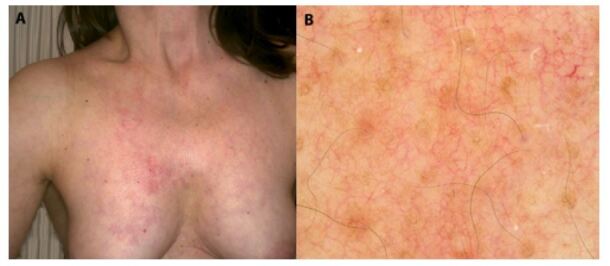
(A) Patches of superficial telangiectasia involving the chest bilaterally. (B) Dermoscopic image of ectatic, tortuous, and thin capillaries.

A 40-year-old woman presented to the dermatology clinic after pregnancy for new-onset of asymptomatic patches of superficial telangiectasia, partially blanchable, mostly arranged unilaterally on the right side of her upper body, including neck, shoulder, and arm. Lesions also involved bilaterally the cervical dermatomes on the chest (Figure 1A). Dermoscopy showed ectatic, tortuous, and thin capillaries (Figure 1B).

Diagnosis of unilateral nevoid telangiectasia (UNT) was made upon clinical and dermoscopic findings.

## Teaching Point

UNT is a rare, nevertheless underdiagnosed, and underreported capillary malformation, associated with increased estrogen levels [1]. It may have a striking appearance, but patients should be reassured about the benignity of the condition [2].
